# Potential links between PCOS/PMOS and Alzheimer disease and related dementias

**DOI:** 10.1210/jendso/bvag206

**Published:** 2026-10-13

**Authors:** Mina Amiri, Armita Kakavand Hamidi, Daniel L Farkas, Richard P Buyalos, Ricardo Azziz

**Affiliations:** Metabolic Disorders Research Center, Endocrinology and Metabolism Molecular-Cellular Sciences Institute, Tehran University of Medical Sciences (TUMS), Tehran 1411713119, Iran; Foundation for Research and Education Excellence, Vestavia, AL 35243, USA; Metabolic Disorders Research Center, Endocrinology and Metabolism Molecular-Cellular Sciences Institute, Tehran University of Medical Sciences (TUMS), Tehran 1411713119, Iran; Obesity and Eating Habits Research Center, Endocrinology and Metabolism Clinical Sciences Institute, Tehran University of Medical Sciences (TUMS), Tehran 1411713119, Iran; Foundation for Research and Education Excellence, Vestavia, AL 35243, USA; Department of Biomedical Engineering, University of Southern California, Los Angeles, CA 90089, USA; Foundation for Research and Education Excellence, Vestavia, AL 35243, USA; Department of Obstetrics and Gynecology, Heersink School of Medicine, University of Alabama at Birmingham (UAB), Birmingham, AL 35249, USA; Foundation for Research and Education Excellence, Vestavia, AL 35243, USA; Department of Medicine, Heersink School of Medicine, UAB, Birmingham, AL 35249, USA; Department of Healthcare Organization and Policy, School of Public Health, UAB, Birmingham, AL 35249, USA; Department of Health Policy, Management, and Behavior, School of Public Health, University at Albany, SUNY, Rensselaer, NY 12144, USA

**Keywords:** polycystic ovary syndrome (PCOS), polyendocrine metabolic ovarian syndrome (PMOS), Alzheimer disease (AD), dementia, cognitive decline, neuroinflammation

## Abstract

**Background:**

Polycystic ovary syndrome/polyendocrine metabolic ovarian syndrome (PCOS/PMOS) is a common endocrine disorder with significant metabolic and reproductive implications. Increasing attention has focused on its potential long-term neurological consequences, including possible links to cognitive impairment and Alzheimer disease (AD), through overlapping biological pathways.

**Methods:**

We conducted a comprehensive PubMed search, from inception through October 2025, identifying studies on cognitive performance, brain structure and function, neurodegenerative biomarkers, and dementia risk in PCOS/PMOS patients.

**Results:**

Evidence supports multiple potential mechanistic pathways that could connect PCOS/PMOS to AD-relevant biology, such as insulin resistance (IR), chronic inflammation, hormonal dysregulation, and cardiovascular dysfunction. These pathways converge on neurodegeneration processes such as impaired Aβ clearance, tau hyperphosphorylation, oxidative stress, and cerebral metabolic deficits. Clinically, PCOS/PMOS patients exhibit mild but reproducible impairments in executive function and memory, alongside structural and functional brain alterations, including hippocampal and white matter changes, and altered network connectivity. However, these findings are influenced by coexisting metabolic dysfunction, and direct epidemiological evidence linking PCOS/PMOS to increased dementia incidence remains limited and inconsistent.

**Conclusion:**

Current evidence indicates that any association between PCOS/PMOS and AD risk is likely mediated by metabolic comorbidities, particularly IR and obesity, rather than an independent effect of PCOS/PMOS itself. However, clinical evidence for increased PCOS/PMOS risk for cognitive vulnerability and dementia remain inconsistent and cognitive benefits from PCOS/PMOS-specific interventions are lacking. Clinical focus should therefore remain on optimizing metabolic health, while longitudinal and interventional studies are required to clarify long-term neurological relationships and outcomes.

Polycystic ovary syndrome (PCOS) recently renamed polyendocrine metabolic ovarian syndrome (PMOS) (1), collectively PCOS/PMOS, affects about 6% to 11% of reproductive-aged women worldwide (2), with prevalence varying by diagnostic criteria (2, 3). Defined by androgen excess, ovulatory dysfunction, and polycystic ovaries (4), PCOS/PMOS is frequently accompanied by metabolic disturbances (4, 5), including insulin resistance (IR) (6, 7), obesity (8-11), and chronic inflammation (12). Collectively, these features increase the risk of type 2 diabetes (T2D) (13, 14), hypertension (15-17), and cardiovascular disease (CVD) (17, 18). Beyond reproductive and metabolic manifestations, PCOS/PMOS is associated with anxiety, depression, and sleep disturbances, suggesting broader effects on brain function (19). These observations have raised interest in potential relationships with cognitive decline and Alzheimer disease (AD) (20, 21).

AD and related dementias represent a growing global health burden, with prevalence projected to exceed 115 million by 2050 (22). These disorders are characterized by progressive cognitive decline (23) arising from multifactorial processes including genetic susceptibility (24, 25), aging (26), biological sex (27, 28), obesity (29), IR (30), neuroinflammation (31), hormonal disturbances (32-35), CVD (36), and oxidative stress (37, 38). Many of these risk factors are prevalent or emerge earlier in PCOS/PMOS.

Emerging evidence suggests PCOS/PMOS patients exhibit alterations across executive function, attention, and memory domains (32, 39-46). Consequently, it has been hypothesized that PCOS/PMOS may increase vulnerability to AD and related dementias (21, 47). Proposed mechanisms include IR, chronic inflammation, and endocrine dysregulation, implicated in neurodegeneration (20, 30, 32, 48-50). However, whether associations reflect PCOS/PMOS intrinsic mechanisms vs coexisting metabolic comorbidities remains unclear. Preliminary interventional studies suggest that modifying key PCOS/PMOS features may improve cognitive performance (51) and normalize abnormal task-elicited brain activation patterns during cognitive or emotional challenges as measured by functional MRI (fMRI) (41, 52), implying such neurological changes may be modifiable.

This review integrates epidemiological, neuroimaging, biomarker, and mechanistic evidence examining links between PCOS/PMOS and cognitive function, brain structure, and AD-related pathways, and distinguishes metabolic comorbidity-mediated effects from PCOS/PMOS-specific effects. We also identify knowledge gaps and priorities for future research.

## Materials and methods

A PubMed search through October 2025 used 2 complementary strategies. Because PMOS is a recently introduced term (1) and most indexed literature still uses PCOS, our search employed PCOS-related terms. First, clinical studies were identified by combining PCOS-related terms (PCOS, “polycystic ovary syndrome,” “polycystic ovarian disease”) with Alzheimer, dementia, cognitive, and neuroimaging terms (“Alzheimer disease,” “dementia, hippocampus,” “prefrontal cortex,” amygdala, “gray matter,” “white matter,” “cognitive function,” “executive function,” “memory, attention,” “functional connectivity,” “brain-derived neurotrophic factor,” BDNF, “brain insulin resistance”). Second, a mechanistic search combined terms for shared pathophysiological pathways (“insulin resistance,” hyperinsulinemia, “chronic inflammation,” “oxidative stress,” “mitochondrial dysfunction,” autophagy, kynurenine, “gut microbiome,” hyperandrogenism, “advanced glycation end products,” “leptin resistance”) with the same cognitive and neurodegenerative terms.

No language or study design restrictions were applied. Clinical studies were included if they assessed cognitive, neuroimaging, biomarker, or dementia-related outcomes in women with PCOS/PMOS; mechanistic studies were included if they examined pathways relevant to both PCOS/PMOS and neurodegeneration. Selection involved title/abstract screening, full-text review, and manual screening of references. When multiple publications originated from the same cohort, the most complete or recent report was selected. Table 1 lists the included clinical studies.

**Table 1:** Summary of clinical studies investigating cognitive function, brain structure, and biomarkers in women with PCOS/PMOS

First author (year)	Study design and duration	Study groups (n)	Key outcomes and measures	Primary findings
Kumari (2025) (51)	Longitudinal interventional pre–post design; 12 weeks (3 cycles) of COC (levonorgestrel 0.15 mg + ethinyl estradiol 30 µg)	PCOS/PMOS: 35 drug-naïve women; no control group	Working memory (auditory/visual digit span), attention (CPT-IP reaction time, random errors), executive function (TMT)	Significant post-treatment improvements in auditory and visual working memory span, faster attention reaction time, fewer random errors, and better executive function; no control group limits causal inference.
Henney (2025) (53)	Prospect. Cohort; Nationwide (UK Biobank)	PCOS/PMOS (n = 1008); age- and BMI-matched controls (n = 5017)	All-cause dementia incidence	No significant association (HR 0.96, 95% CI 0.32-2.89). Subgroup analyses by phenotype (hyperandrogenic vs normoandrogenic) also nonsignificant.
Redkar (2025) (39)	Cross-sectional	PCOS/PMOS (n = 101); controls (n = 72) age was similar in both groups but weight was significantly higher in PCOS/PMOS group	Focused/divided attention (Flanker, Posner tasks)	Slower reaction times (557 vs 463 ms) and more errors in focused attention in PCOS/PMOS group.
Chen (2025) (35)	Case-control Rotterdam	PCOS/PMOS (n = 76); age-, sex-, BMI-, and ethnicity-matched controls (n = 68)	Gray matter volume, cortical thickness (MRI); DHEAS levels	Milder age-related gray matter decline in PCOS/PMOS. High DHEAS revealed neuroprotective effect.
Butler (2024) (20)	Cross-sectional	Nondiabetic PCOS/PMOS (n = 137); age-matched controls (n = 97) analyses adjusted for age, hyperandrogenism, obesity, IR, and fasting glucose	Plasma AD biomarkers (APP, APCS, SNCA, ApoE, and FN)	Elevated plasma levels of AD-related proteins (APP, APCS, ApoE, and FN) and reduced α-synuclein (SNCA) in PCOS/PMOS women, a pattern strikingly similar to that observed in type 2 diabetes. When stratifying according to hyperandrogenemia, with APCS, ApoE and ApoA1 higher in hyperandrogenemia, while PAPPA was decreased in hyperandrogenemia. There was no difference seen when stratifying according to obesity, IR, fasting glucose, or age.
Butler (2024) (54)	Cross-sectional	Nonobese PCOS/PMOS (n = 24); age- and BMI-matched nonobese, noninsulin-resistant, and nondiabetic controls (n = 24)	Plasma Dementia Proteins (APP, SNCA, APCS, MAPT, and ApoE)	Only APCS significantly elevated. Overall biomarker expression lower than in obese PCOS/PMOS.
Vine (2024) (21)	Retrospect. cohort	PCOS/PMOS (n = 16 531); age-matched controls (n = 49 335)	Dementia prevalence, age at onset	Higher dementia prevalence in PCOS/PMOS (0.48% vs 0.22%); younger onset age (43 vs 62 years).
Huddleston (2024) (55)	Prospective cohort; 30-year follow-up (CARDIA)	PCOS/PMOS (n = 66); matched controls (n = 841) Groups matched for age, BMI, smoking/drinking status, and income. Analyses adjusted for age, race, education, and study center	Cognitive function (Stroop, RAVLT), brain MRI (DTI white matter integrity)	Lower cognitive scores and reduced white matter integrity in PCOS/PMOS group at midlife.
Showkath et al (2023) (56)	Case-control	PCOS/PMOS without comorbidities (n = 37); age-matched healthy controls (n = 30)	Global cognition (MoCA), EEG, P300 amplitude/latency	Lower MoCA scores, abnormal EEG power, reduced P300 amplitude/increased latency.
Sukhapure et al (2022) (57)	Longitudinal, naturalistic open-label study; 12 weeks of individualized standard pharmacotherapy (predominantly spironolactone, n = 22; oral contraceptives, n = 16)	PCOS/PMOS: 33 treated; controls: 40 age-matched untreated healthy	Depression and anxiety (HADS); cognitive function (verbal and visuospatial learning/memory, psychomotor speed, attention, executive function)	Depression and anxiety symptoms improved significantly (moderate effect sizes). Cognitive improvements occurred within the PCOS/PMOS group but did not differ significantly from controls, indicating practice effects rather than treatment-specific cognitive enhancement.
Huddleston et al (2022) (58)	Cross-sectional; phenotype-based	HA-PCOS/PMOS (n = 31); NA-PCOS/PMOS (n = 17)	Executive function, memory, verbal/perceptual reasoning	HA-PCOS/PMOS had lower executive function, especially with coexisting IR.
Li et al (2020) (46)	Case-control	Newly diagnosed PCOS/PMOS (n = 41); age, education, sex, and handedness-matched healthy controls (n = 41)	Cognition, emotion, brain activity (ALFF/FC), hormones	Cognitive deficits and altered brain connectivity correlated with insulin and LH levels.
Jarrett (2019) (40)	Cross-sectional	PCOS/PMOS (n = 40); controls (n = 40) analyses adjusted for BMI	Cognitive domains (working memory, manual dexterity, perceptual speed, visuospatial ability), metabolic markers (HbA1c)	Reduced visuospatial ability linked to higher HbA1c, not to PCOS/PMOS status or sex steroids.
Franik (2019) (59)	Cross-sectional	Normal-weight PCOS/PMOS patients (n = 55)	Executive function (trail making, stroop), verbal fluency, processing speed, hormonal/metabolic markers	High insulin/androstenedione linked to worse function; free testosterone to better speed.
Lai (2019) (60)	Case-control	Newly diagnosed PCOS/PMOS (n = 21); controls (n = 27)	Brain activity (ALFF), functional connectivity (FC), LH, and FSH	Decreased frontal lobe FC in PCOS/PMOS, negatively correlated with LH and LH/FSH ratio.
Saydam (2017) (61)	Case-control; obese/lean	Obese/lean PCOS/PMOS vs obese and lean groups BMI-matched; (Total n = 40; 10 lean PCOS/PMOS, 10 lean controls, 10 obese PCOS/PMOS, 10 obese controls)	Brain Structure (total brain volume, gray matter volume), regional gray matter volumes MRI	Obese PCOS/PMOS: reduced total brain and hippocampal volume. Lean PCOS/PMOS: reduced amygdala volume.
Sundararaj (2017) (62)	Case-control	PCOS/PMOS (n = 10); controls (n = 10) participants screened to exclude comorbidities	Auditory working memory (digit span tasks), hearing	Poorer performance on auditory working memory tasks and high-frequency hearing in PCOS/PMOS.
Soleman (2016) (41)	Longitudinal interventional fMRI; 16-week antiandrogen treatment (cyproterone acetate + ethinyl estradiol)	HA-PCOS/PMOS: 14 (9 completed); controls: 20 untreated ovulatory (16 completed); age/IQ-matched	Working memory, brain activation (fMRI), accuracy	Greater parietal activation at baseline despite similar performance in PCOS/PMOS. Post-treatment, group brain differences resolved and PCOS/PMOS accuracy improved under high load.
Guoqing (2016) (63)	Case-control; postmenopausal	Postmenopausal PCOS/PMOS (n = 70); age and hypertension prevalence matched controls (n = 140)	Cerebrovascular disease (WMLs, SCIs on MRI)	Higher prevalence of WMLs (40% vs 22%) and SCIs (17% vs 7%) in PCOS/PMOS group.
Rees (2016) (42)	Cross-sectional	PCOS/PMOS (n = 18); age, BMI, and IQ-matched controls (n = 18)	White matter microstructure (DTI), cognitive domains	Altered white matter (corpus callosum) and subtle cognitive reductions across domains.
Castellano (2015) (43)	Case-control; PET-MRI	PCOS/PMOS (n = 7); age, BMI, and education-matched controls (n = 11)	Cerebral glucose metabolism (CMRglu), HOMA2-IR	9-14% lower CMRglu in PCOS/PMOS, inversely correlated with IR.
Marsh (2013) (52)	Case-control pilot; 16-week open-label metformin (1500 mg/day)	IR-PCOS/PMOS: 7; controls: 5 noninsulin-resistant, age-restricted, unmatched for BMI/androgens	Limbic activation during emotional processing (fMRI); mu-opioid receptor availability (PET); mood (positive affect, trait anxiety)	IR-PCOS/PMOS showed greater limbic activation and trended toward lower positive affect and higher anxiety vs controls. Postmetformin, limbic activation normalized and no longer differed from controls. Limbic activation correlated positively with mu-opioid binding potential.
Barry (2013) (64)	Cross-sectional	PCOS/PMOS (n = 69); controls (n = 41) (not age- or BMI-matched; fertility-status–matched controls; age and BMI adjusted in subgroup analyses)	Mental rotation (3D), correlation with testosterone	Higher mental rotation scores in PCOS/PMOS; performance positively correlated with testosterone.
Barnard (2007) (45)	Cross-sectional; internet-based	PCOS/PMOS (n = 135); controls (n = 322) (not matched for age, BMI, or depression; age-adjusted analyses; stratified by depression and antiandrogen use)	Multiple cognitive domains (mental rotation, spatial location, reaction time, and word recognition)	No masculinized profile; PCOS/PMOS showed slower reaction times and reduced word recognition.
Schattmann (2007) (32)	Case-control; cross-sectional	PCOS/PMOS (n = 29); age- and education-matched controls (n = 22) (BMI, IQ, and mood comparable; diabetes excluded; no statistical adjustment)	Cognitive domains (verbal fluency, verbal memory, manual dexterity, visuospatial working memory, spatial skills, and perceptual speed)	Deficits in verbal fluency, memory, manual dexterity, and visuospatial working memory in PCOS/PMOS.
Schattmann & Sherwin (2007) (65)	RCT; 14-week antiandrogen (cyproterone acetate 50 mg + ethinyl estradiol 35-50 μg) vs placebo	PCOS/PMOS-HA: intervention (antiandrogen + estrogen): n = 8; placebo: n = 11; randomized, groups matched at baseline	Verbal fluency (COWAT), verbal memory, visuospatial ability, visuospatial working memory, manual dexterity, perceptual speed	Testosterone suppression significantly improved verbal fluency on 1 trial; no treatment effects on visuospatial ability, verbal memory, manual dexterity, or perceptual speed.

Abbreviations: AD, Alzheimer disease; ALFF, amplitude of low-frequency fluctuations; APCS, amyloid P component, serum; ApoE, apolipoprotein E; APP, amyloid precursor protein; CMRglu, cerebral metabolic rate of glucose; COC, combined oral contraceptive; CPT-IP, continuous performance test-identical Pairs; DHEAS, dehydroepiandrosterone sulfate; DTI, diffusion tensor imaging; EEG, electroencephalography; FC, functional connectivity; fMRI, functional magnetic resonance imaging; FN, fibronectin; HA-PCOS/PMOS, hyperandrogenic PCOS/PMOS; HbA1c, glycated hemoglobin; HOMA2-IR, homeostasis model assessment of insulin resistance; HR, hazard ratio; IR-PCOS/PMOS, insulin-resistant PCOS/PMOS; LH, luteinizing hormone; long., longitudinal; MoCA, Montreal Cognitive Assessment; MRI, magnetic resonance imaging; NA-PCOS/PMOS, normoandrogenic PCOS/PMOS; PCOS/PMOS, polyendocrine metabolic ovarian syndrome (formerly polycystic ovary syndrome or PCOS); PET, positron emission tomography; RAVLT, Rey Auditory Verbal Learning Test; RCT, randomized controlled trial; SCI, silent cerebral infarct; SNCA, α-synuclein; T2D, type 2 diabetes; TMT, trail making test; WM, working memory; WML, white matter lesion.

## Results

### Pathophysiology of PCOS/PMOS: a complex interplay of hormones and metabolism

PCOS/PMOS involves complex genetic, endocrine, metabolic, and neural interactions. A core defect in 75% to 95% of patients (66) is IR with compensatory hyperinsulinemia, which stimulates ovarian and adrenal hyperandrogenism by enhancing luteinizing hormone (LH) action and steroidogenesis. Theca cells intrinsically overproduce androgens (67). Insulin suppresses SHBG, increasing bioactive androgens that worsen IR (68). This imbalance disrupts the hypothalamic–pituitary–ovarian (HPO) axis, where elevated androgens increase GnRH frequency (69), elevating LH:FSH ratio and perpetuating anovulation (3). Preclinical studies indicate hyperandrogenism alters arcuate nucleus signaling, disrupting GABA, kisspeptin, and proopiomelanocortin pathways (47).

Tissue-specific metabolic dysfunction in adipose tissue, skeletal muscle, and liver contributes to metabolic disturbances that increase T2D and cardiovascular risk. Adipose dysfunction contributes to IR through impaired glucose uptake, altered adipokine secretion, and ectopic lipid deposition (70). Androgen excess impairs adipose function and promotes visceral fat, amplified by the concurrent obesity. A meta-analysis of 58 population-based studies (85 956 participants from 24 countries) reported a modest positive association between obesity and PCOS/PMOS prevalences (7).

In skeletal muscle, insulin signaling is impaired; a contributing factor appears to be excessive serine phosphorylation of insulin receptor (71). The serine phosphorylation defect persists in cultured fibroblasts, indicating a constitutive element, but it does not correlate with circulating androgens, and GnRH agonist suppression of hyperandrogenism does not improve insulin sensitivity (71). However, IR in PCOS/PMOS is now recognized as a complex, multifactorial condition that involves multiple tissues and overlaps with the IR observed in obesity and T2D (6, 72).

In the liver, reduced SHBG production links to metabolic dysfunction-associated steatotic liver disease (73). Hepatic SHBG reduction relates more closely to IR and adiposity than androgen levels (73). Whether severity of tissue-specific abnormalities is influenced by age at PCOS/PMOS diagnosis remains unknown. Combined with chronic inflammation (74) and increased sympathetic overactivity (75), these changes can increase the risk of T2D and CVD.

Central nervous system alterations in PCOS/PMOS include reduced global and regional gray matter volumes, altered white matter microstructure, decreased functional connectivity within cognitive networks, and diminished cerebral glucose metabolism, as documented by structural and functional neuroimaging studies. These brain changes have been linked to the neuroendocrine dysfunction, cognitive deficits (particularly in executive function and memory), and the elevated rates of anxiety and depression observed in affected women (47).

The syndrome is associated with anxiety and depression, and sleep disturbances (76, 77), with evidence from preclinical models suggesting that fetal programming by maternal androgens plays a role (78). Daily administration of dihydrotestosterone (250 µg) to pregnant mice during late gestation (embryonic day 16.5-18.5) produced transgenerational PCOS/PMOS-like traits in female offspring. PCOS/PMOS patients have ∼3-fold higher circulating androgens during pregnancy, although the threshold for triggering such reprogramming in human pregnancy is unknown (78). Further, limited evidence suggests maternal PCOS/PMOS may associate with an increased risk of neurodevelopmental disorders in offspring, including autism spectrum disorder and attention-deficit/hyperactivity disorder (79-82).

Thus, PCOS/PMOS is a systemic disorder in which hormonal and metabolic dysfunction may affect neurological health. Lean women with PCOS/PMOS exhibit intrinsic IR independent of obesity (83, 84), whereas concurrent obesity amplifies IR and inflammation, producing a more severe metabolic phenotype (54, 70). Cognitive and brain structural differences observed in PCOS/PMOS appear to be predominantly mediated by these metabolic comorbidities: deficits in visuospatial ability correlate with glycemic control rather than androgen levels (40) and circulating AD-associated protein profiles are largely normal in PCOS/PMOS patients who are metabolically healthy (60). However, neuroimaging studies have identified functional connectivity changes that correlate with LH concentrations independently of metabolic parameters (58), suggesting that hormonal factors may exert additional, albeit more modest, direct effects on the brain.

### Clinical evidence: cognitive function, brain structure, and dementia

Table 1 summarizes the key clinical studies examining cognitive performance, neuroimaging, and dementia outcomes in PCOS/PMOS.

#### Cognitive function and PCOS/PMOS

Multiple studies have examined cognitive performance in PCOS/PMOS patients. Deficits are evident in executive function (44, 62), and attention (39), with slower reaction times and increased errors on focused attention tasks. Memory impairments, including auditory working memory (56) and verbal memory (32), have also been reported. A case-control EEG-ERP study (37 PCOS/PMOS, 30 controls) reported reduced P300 amplitude and increased latency, indicating less efficient brain activity, though the sample was modest, and comorbidities were excluded (61). An internet-based neuropsychological study (135 PCOS/PMOS, 322 controls) found women with PCOS/PMOS do not exhibit a male-typical pattern (eg, enhanced visuospatial performance or poorer verbal fluency), but demonstrate broad, subtle declines in processing speed and word recognition (45). Reduced visuospatial ability linked to higher HbA1c levels, independent of PCOS/PMOS status (40).

#### Brain structure and function and PCOS/PMOS

Despite modest sample sizes, neuroimaging studies provide a biological substrate for these cognitive findings in PCOS/PMOS. MRI studies reveal both vulnerability and possible neuroprotection: a small BMI-matched study (n = 40) found reduced hippocampus volume in obese PCOS/PMOS and reduced amygdala volume in lean PCOS/PMOS (55), while larger case-control study (76 PCOS/PMOS and 68 controls) attributed milder age-related gray matter decline to elevated DHEAS levels (35). White matter microstructure alterations, indicating compromised connectivity, have been documented in both young and middle-aged PCOS/PMOS patients (42, 85). fMRI studies demonstrate decreased connectivity within networks supporting complex cognition (46, 58) and abnormal brain activation patterns during cognitive tasks (41, 46). A PET-MRI study in normal-weight women (7 PCOS/PMOS and 11 controls) identified reduced cerebral glucose metabolism correlating with IR (43).

#### Dementia, Alzheimer disease and PCOS/PMOS

AD is the most common form of dementia, characterized by memory impairment and cognitive deterioration that disrupts daily functioning (29). AD and related dementias result from molecular, cellular, and systemic abnormalities that impair brain structure and function (29, 86). The primary pathological hallmarks are extracellular β-amyloid (Aβ) plaques and intracellular neurofibrillary tangles (NFTs) of hyperphosphorylated tau (86). These processes lead to widespread brain atrophy, particularly in hippocampus and cortex, underlying progressive cognitive decline (87, 88). The multifactorial nature of these disorders underscores the need for integrated approaches to pathogenesis, prevention, early diagnosis, and treatment (89).

Sex is an important determinant of AD risk. Women over 65 have 2- to 3-fold higher AD risk than men, a disparity not fully explained by longevity (27, 90) and likely reflecting interactions among genetic, hormonal, medical, psychosocial, and behavioral factors (27, 28, 91, 92). Proteomic analyses reveal sex-specific brain protein expression patterns and sex-by-trait interactions in AD, implicating estrogen signaling, inflammation, and mitochondrial pathways (93). Women demonstrate heightened Aβ-dependent p-tau secretion, potentially driven by stronger ApoE4-related risk and microglial activation (94).

These biological vulnerabilities interact with modifiable life experiences; for instance, among APOE4 carriers, higher occupational attainment correlated to cognitive benefit in women but poorer cognition in men (95), suggesting sex-specific operation of cognitive reserve. Early life programming may establish long-term vulnerabilities. A longitudinal study of 204 offspring followed from gestation to early midlife (∼50 years) demonstrated that prenatal exposure to a proinflammatory maternal environment is associated with sex-dependent alterations in memory circuitry and a hyperimmune state persisting into midlife (96). These findings indicate that female-predominant conditions such as PCOS/PMOS warrant investigation as potential AD risk contributors. These data also suggest that higher androgens independently do not increase AD risk.

In PCOS/PMOS, emerging studies report elevated plasma levels of amyloid precursor protein (APP) and Apolipoprotein E (ApoE), proteins central to AD pathology, with patterns resembling those observed in AD and T2D. APP serves as the precursor to Aβ, while APCS stabilizes amyloid fibrils, suggesting a peripheral milieu permissive to amyloid aggregation. Positive correlations between APCS and proinflammatory cytokines mirror the bidirectional amplification of inflammation and amyloid pathology observed in AD. This biomarker profile is more pronounced in obese PCOS/PMOS (20), whereas in rigorously matched nonobese, noninsulin-resistant PCOS/PMOS cohorts, only serum amyloid P component (APCS) is modestly elevated (60).

APCS is detected in plaques and the NFTs characteristic of AD (97) and may play an important role in the decreased proteolysis of Aβ deposits, resulting in further plaque formation (98). However, the specificity of this amyloid signature to PCOS/PMOS appears limited. In rigorously matched studies of nonobese, noninsulin-resistant PCOS/PMOS patients, only APCS, but not the broader amyloid protein profile, was elevated (60). This indicates that full amyloid pathway dysregulation likely depends on metabolic dysfunction rather than PCOS/PMOS per se (53). Direct evidence of brain amyloid deposition in PCOS/PMOS is lacking, and existing studies rely on peripheral biomarkers. Thus, chronic inflammation in PCOS/PMOS likely facilitates rather than initiates neurodegenerative processes, particularly in metabolically unhealthy phenotypes.

The most direct evidence linking PCOS/PMOS to clinical dementia derives from studies examining dementia incidence. These data are limited and inconsistent. A retrospective cohort study, involving 16 531 patients with PCOS/PMOS and 49, 335 age-matched controls, reported higher dementia prevalence in PCOS/PMOS (0.48% vs 0.22%) and a younger mean age of onset (43 vs 62 years) (21). However, a prospective UK biobank analysis of 1008 women with PCOS/PMOS compared with 5017 age- and BMI-matched controls found no significant association between PCOS/PMOS and all-cause dementia incidence, even when stratified by androgenic phenotype (65). These divergent findings highlight the critical influence of study design, diagnostic criteria, and, most importantly, ability to adjust for metabolic confounders. Studies that control for BMI and IR demonstrate marked attenuation or absence of increased dementia risk (65), suggesting that metabolic comorbidities, rather than PCOS/PMOS itself, drive any observed association.

#### Cognition and intervention studies in PCOS/PMOS

Intervention studies examining whether modifying PCOS/PMOS features influences cognitive function remain preliminary, characterized by small sample sizes, heterogeneous designs, and inconclusive findings. A 16-week longitudinal study of 14 hyperandrogenic PCOS/PMOS patients receiving cyproterone acetate plus ethinyl estradiol found that, compared with the 20 untreated controls, patients exhibited greater parietal activation during working memory despite comparable task performance, suggesting compensatory neural inefficiency. After treatment, group differences in brain activation resolved and accuracy under high load improved, although hormonal suppression did not directly correlate with neural changes (41). A 14-week RCT in 19 women with hyperandrogenic PCOS/PMOS found that hormonal contraceptives containing antiandrogenic progestins significantly reduced free testosterone but improved verbal fluency on only 1 trial, with no effects on visuospatial ability, verbal memory, manual dexterity, or perceptual speed (57).

A 12-week pre–post study of 35 drug-naïve women treated with a levonorgestrel-containing oral contraceptive found broad post-treatment gains in working memory, attention, and executive function; however, the absence of a control group limits causal inference (51). In insulin-resistant PCOS/PMOS, a 16-week pilot case-control study demonstrated that metformin therapy normalized exaggerated limbic activation during emotional processing, with changes correlating with mu-opioid receptor availability (52). Finally, a 12-week naturalistic study of individualized pharmacotherapy (predominantly spironolactone and oral contraceptives) in 33 women found significant mood improvements but no cognitive gains beyond practice effects when compared with 40 healthy controls (99).

#### Summary of clinical evidence

Several consistent findings emerge from this body of evidence. First, PCOS/PMOS patients exhibit mild, reproducible deficits in executive function and attention, accompanied by reduced hippocampal volume, white matter microstructural alterations, and decreased cerebral glucose metabolism. Second, these differences are consistently attenuated or absent in metabolically healthy PCOS/PMOS subgroups, particularly those without obesity or IR. Third, direct epidemiologic evidence linking PCOS/PMOS to incident dementia is inconclusive: a positive retrospective study is contradicted by a prospective cohort that found no association after metabolic adjustment. Fourth, intervention studies suggest some PCOS/PMOS treatments may improve specific cognitive domains or normalize brain activation patterns, but findings are inconsistent, effect sizes are modest, control groups are often lacking, and no study has examined dementia as an endpoint.

Overall, PCOS/PMOS may be associated with mild cognitive and neuroanatomical differences, but these do not clearly increase dementia risk in the absence of metabolic comorbidity. Metabolic dysfunction, particularly obesity and IR, appears to be the primary mediator of any observed association between PCOS/PMOS and adverse brain outcomes.

### Potential shared pathways between PCOS/PMOS and cognitive impairment disorders

The clinical evidence indicates that any association between PCOS/PMOS and adverse brain outcomes is predominantly driven by metabolic comorbidities, especially obesity and IR. We first examine shared metabolic and vascular pathways (obesity, IR, dyslipidemia, cardiovascular dysfunction, sleep disturbances, and gut microbiome dysbiosis), followed by PCOS/PMOS-related hormonal dysregulation (where evidence for independent contribution is less robust), and finally intersecting downstream mechanisms (genetic/epigenetic factors, chronic inflammation, oxidative stress, autophagy, and tryptophan–kynurenine pathway dysregulation).

Many of these pathways are not unique to PCOS/PMOS but are central to obesity, IR, and T2D, conditions highly prevalent in PCOS/PMOS. Conversely, certain PCOS/PMOS intrinsic features, such as hyperandrogenism and chronic HPO axis dysregulation, may contribute directly to cognitive vulnerability even without pronounced metabolic dysfunction. These interconnected processes create a systemic milieu capable of influencing brain structure and function (Fig. 1). Whether these changes ultimately increase susceptibility to AD and related dementias remains uncertain; current data suggest a phenotype-dependent association rather than a uniform neurodegenerative trajectory (21, 47). These pathways are examined in detail below.

**Figure 1:**
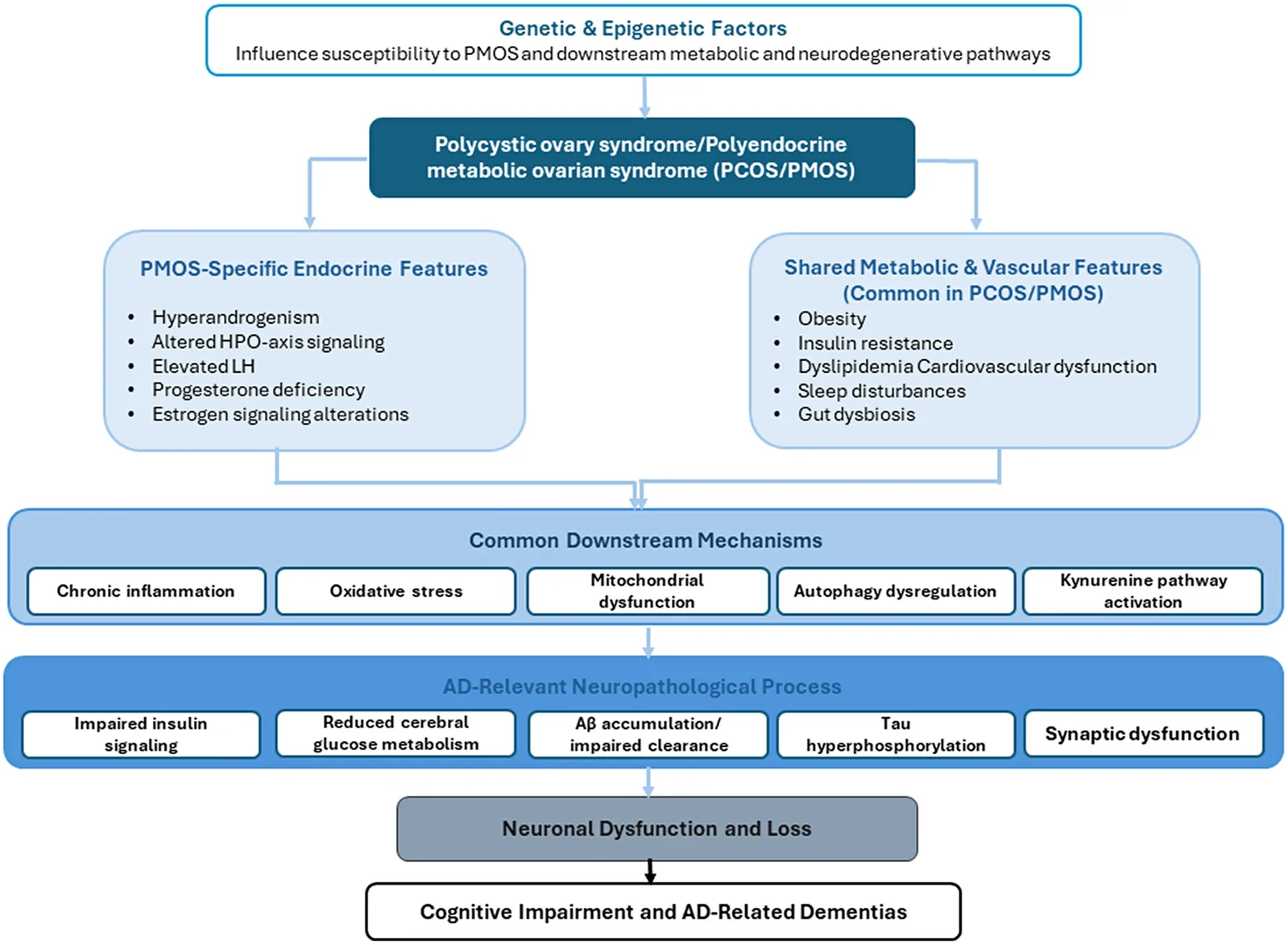
Proposed pathways linking polycystic ovary syndrome (PCOS/PMOS) to cognitive impairment and Alzheimer disease (AD)-related dementias. Genetic and epigenetic factors may influence susceptibility to PCOS/PMOS and its downstream metabolic and neurological consequences. PCOS/PMOS is characterized by endocrine abnormalities, including hyperandrogenism, hypothalamic–pituitary–ovarian (HPO) axis dysregulation, elevated luteinizing hormone (LH), progesterone deficiency, and altered estrogen signaling. In parallel, individuals with PCOS/PMOS frequently exhibit metabolic and vascular comorbidities, including obesity, insulin resistance (IR), dyslipidemia, cardiovascular dysfunction, sleep disturbances, and gut microbiome dysbiosis. These endocrine and metabolic factors converge on several shared downstream mechanisms, including chronic inflammation, oxidative stress, mitochondrial dysfunction, autophagy dysregulation, and activation of the kynurenine pathway. Collectively, these processes may contribute to AD-relevant neuropathological changes, including impaired insulin signaling, reduced cerebral glucose metabolism, β-amyloid (Aβ) accumulation and impaired clearance, tau hyperphosphorylation, and synaptic dysfunction, ultimately leading to neuronal dysfunction, cognitive impairment, and dementia. Current evidence suggests that metabolic comorbidities, particularly obesity and IR, are the principal mediators of any potential association between PCOS/PMOS and adverse brain outcomes, whereas the independent contribution of PCOS/PMOS-specific endocrine abnormalities remain less certain.

#### Obesity

Obesity is more prevalent in PCOS/PMOS but affects <50% of women (100). The concurrent presence of obesity appears to be a central modifier of neurological risk in PCOS/PMOS (8), independently associated with AD and dementias (29, 101). Preclinical studies indicate that obesity may increase Aβ burden through enhanced APP expression in adipose tissue and increased circulating Aβ, which crosses the blood brain barrier via the receptor for advanced glycation end products, promoting neuronal stress (102). Obesity-related reductions in brain-derived neurotrophic factor (BDNF) and dyslipidemia further enhance Aβ production by stimulating β- and γ-secretase activity (103, 104). However, direct evidence linking adipose-derive Aβ to brain pathology in human PCOS/PMOS remains limited.

#### Metabolic dysfunction

Diabetes significantly increases dementia risk, including a 56% increased AD risk (105). Molecular parallels exist between IR in PCOS/PMOS and central AD mechanisms (53) where impaired insulin signaling promotes tau hyperphosphorylation, amyloidogenic APP processing, and reduced Aβ clearance through competition for insulin-degrading enzyme (59, 106-110). IR disrupts neuronal glucose uptake and energy metabolism, particularly in the hippocampus and cortical regions, leading to synaptic dysfunction and bioenergetic stress similar to T2D-associated cognitive decline (88, 111). PET-MRI studies in young PCOS/PMOS patients demonstrate reduced cerebral glucose metabolism correlating with IR (43), indicating early neural metabolic stress.

Chronic hyperglycemia worsens this deficit by forming advanced glycation end products (AGEs), which modify Aβ and tau, enhancing their aggregation and promoting the oxidative stress and neuroinflammation (112, 113). Nevertheless, human evidence linking PCOS/PMOS-specific IR, as distinct from the well-established association between diabetes and AD, to AD pathology remains indirect. Most biomarker and imaging studies are cross-sectional, modestly powered, and often lack stratification by metabolic phenotype. Importantly, AD-like biomarker profiles are markedly attenuated in nonobese, insulin-sensitive PCOS/PMOS populations (60), reinforcing that IR/glucose intolerance, rather than PCOS/PMOS diagnosis, is the dominant driver of AD-related mechanisms.

#### Dyslipidemia

Dyslipidemia in PCOS/PMOS, typically low HDL and elevated triglycerides (114, 115), mirrors lipid abnormalities in AD, where reduced HDL and apolipoprotein A-I contribute to neuroinflammation and impaired Aβ clearance (101) and APOE ε4 disrupts cholesterol trafficking and amyloid clearance (116-118). PCOS/PMOS-related lipid disturbances may therefore converge with AD pathways involving oxidative stress, mitochondrial dysfunction, and neuroinflammation (119, 120), but are heavily influenced by the concomitant obesity and IR.

#### Autonomic and cardiovascular dysfunction

Autonomic dysregulation and vascular impairment may link PCOS/PMOS to cognitive decline, particularly relevant given women's heightened vulnerability to dementia from cardiovascular risk factors like hypertension, which are highly prevalent in PCOS/PMOS (15, 121); midlife hypertension confers greater dementia risk in women than men (122). PCOS/PMOS features persistent sympathetic overactivity (123), correlating with IR (124). This imbalance, combined with metabolic risk factors (36), may drive endothelial dysfunction (123), increased left ventricular mass (125), accelerated atherosclerosis (126), and a prothrombotic state (127).

However, many of these abnormalities overlap with obesity and diabetes, complicating attribution to PCOS/PMOS per se. Cerebrally, PCOS/PMOS patients demonstrate higher prevalence of white matter lesions and silent cerebral infarcts (128), consistent with chronic microvascular injury. Most available studies are cross-sectional and lack comprehensive adjustment for lifetime metabolic exposure. Associations between heart rate variability and cognitive performance support a brain–heart axis (129), although whether autonomic dysfunction precedes cognitive changes or reflects metabolic changes is unclear. Autonomic and cardiovascular dysfunction likely represents an indirect pathway through shared cardiometabolic vulnerabilities.

#### Sleep disturbances

Sleep disturbances regulates glymphatic Aβ clearance and is a modifiable AD risk factor (130). Sleep deprivation promotes Aβ accumulation bidirectionally (131). PCOS/PMOS is associated with high prevalence of sleep disturbances, partly attributable to obesity (132), and linked to hyperandrogenism and IR (77). Chronic sleep disruption thus represents a plausible but indirect pathway for impaired Aβ clearance. These mechanisms are shared across obesity, diabetes, and PCOS/PMOS, limiting their specificity (29). Importantly, epidemiological studies controlling for BMI consistently demonstrate attenuated or absent dementia risk in PCOS/PMOS (65), underscoring obesity as a primary mediator.

#### Gut microbiome dysbiosis

The gut microbiota regulates tryptophan metabolism, intestinal permeability, immune activation, inflammation, and insulin sensitivity (133). PCOS/PMOS is characterized by gut dysbiosis, including reduced microbial diversity (134) and lower abundance of butyrate-producing genera such as Roseburia, even in lean individuals (135). Obesity independently promotes similar dysbiosis patterns, increasing lipopolysaccharide (LPS) translocation, metabolic endotoxemia, systemic inflammation, and IR (136-138).

Hyperandrogenism may also contribute to this risk. Androgen levels inversely correlate with microbial biodiversity in lean and obese PCOS/PMOS patients (134, 135, 139). PCOS/PMOS-specific compositional alterations have been observed even after accounting for body weight (137). The combination of obesity- and androgen-related dysbiosis is associated with the lowest bacterial diversity and most pronounced metabolic dysfunction (139). Enrichment of LPS-producing bacteria elevates circulating LPS, promoting inflammation and IR (135, 140), while gut–brain axis disruption in PCOS/PMOS is linked to altered DNA methylation of *FKBP5* (139). Elevated inflammation and LPS provide a plausible link to mood disorders and neuroinflammation (141). However, direct evidence connecting PCOS/PMOS- or obesity-related gut dysbiosis to AD pathology is currently lacking.

#### HPO axis and sex hormone alterations

Although metabolic disturbances are likely the primary drivers of neurological risk, certain PCOS/PMOS intrinsic hormonal features may also contribute, though evidence is less robust. Lifelong HPO axis dysregulation is a defining PCOS/PMOS feature (142) and influences brain health in a sex-specific manner (34), creating hormonal milieu distinct from obesity or diabetes alone (32, 63, 143). Chronic anovulation results in relative progesterone and reduced expression of steroidogenic acute regulatory (StAR) protein, a key cholesterol transporter for steroidogenesis (144).

Preclinical models link reduced hippocampal StAR to diminished neuroprotective neurosteroids and enhanced Aβ deposition (49, 145). Prolonged progesterone deficiency in PCOS/PMOS may therefore deprive the brain of neuroprotective signaling (33, 146), although in vivo evidence in PCOS/PMOS remains limited. Altered kisspeptin neuronal activity, a key regulator of the HPO axis in PCOS/PMOS and in normal individuals, have been postulated to influence amyloid-related processes (147, 148).

Hyperandrogenism, a core PCOS/PMOS feature (149), is associated with poorer cognitive performance via neuroinflammation and IR interactions (32). Androstenedione relates to worse executive function, while free testosterone may exert domain specific effects on processing speed (150). Hyperandrogenism also associates with disturbances in the tryptophan catabolite pathway (151). Elevated DHEAS, particularly in younger women has been linked to slower brain aging and potentially protective effects (35). Most studies are cross-sectional and rely on peripheral hormone measurements, limiting causal inference.

Estrogen signaling is also impaired in PCOS/PMOS; hyperestrogenism from precursors androgens and aromatase activity in obesity (152) may lead to atypical neural estrogen signaling, and a U-shaped association between lifetime estrogen exposure and dementia risk suggests complex effects (153). Elevated LH, common in nonobese PCOS/PMOS, correlates with plasma Aβ (154, 155) and induces spatial memory impairments in experimental models (156), potentially via BDNF-dependent mechanisms (157), though clinical evidence linking chronic LH elevation in PCOS/PMOS to amyloid deposition or neurodegeneration is limited. In summary, PCOS/PMOS-associated HPO disruption may influence neural physiology but is unlikely to independently drive neurodegeneration. Longitudinal studies incorporating phenotype-stratified endocrine profiling, neuroimaging, and cognitive outcomes are needed to clarify their long-term implications.

#### Genetic and epigenetic predisposition

AD and PCOS/PMOS have distinct genetic architectures. AD involves rare mutations in APP, PSEN1, and PSEN2, and common variants in over 20 genes (101), with APOE ε4 being the strongest risk factor (158-160). Genome-wide association studies (GWAS) implicate pathways related to immune regulation, endocytosis, and lipid metabolism (161).

PCOS/PMOS is a highly polygenic, with GWAS identifying loci in gonadotropin signaling and insulin secretion (162, 163). Variations in mitochondrial DNA have also been associated with PCOS/PMOS, suggesting a role for impaired cellular energy metabolism and oxidative stress in disease pathogenesis (164). Despite largely nonoverlapping risk loci, several shared molecular pathways include dysregulated noncoding RNAs (GAS5 and steroid receptor RNA activator) implicated in IR and inflammation (165, 166). The SET protein is overexpressed in PCOS/PMOS ovaries contributing to hyperandrogenism (167, 168) and is also upregulated in the AD brain, where it inhibits PP2A leading to tau hyperphosphorylation (169, 170). Dysregulation of the SET/PP2A axis therefore represents a potential convergent mechanism, although direct genetic linkage between PCOS/PMOS and AD remains unestablished.

A common mechanistic feature shared between AD and PCOS/PMOS is the dysregulated synthesis of StAR protein, a key mitochondrial enzyme that limits the progesterone biosynthesis rate (144). StAR deficiency is observed in both PCOS/PMOS (171) and AD brain (145), linked to elevated LH and heightened kisspeptin activity (147, 148). Further, epigenetic dysregulation may represent a shared mechanistic layer linking PCOS/PMOS and AD. The SET (SE Translocation) protein, a key epigenetic regulator, is overexpressed in both PCOS/PMOS theca cells and AD neurons. SET overexpression is driven by the transcription factor ZFX, suggesting persistent epigenetic dysregulation across tissues (25).

The association between PCOS/PMOS and AD appears to be mediated primarily by chronic inflammation, IR, and hormonal dysregulation, further influenced by PCOS/PMOS heterogeneity and comorbidities like depression and CVD (65, 148). Current evidence is limited by methodological inconsistencies, including variable study durations, small sample sizes, and diverse cognitive assessment tools, which complicate the detection of subtle associations (20, 60, 85, 172), and Mendelian randomization studies have not yet established causality (173). Future research should prioritize large prospective cohorts, phenotype-specific genetic analyses in nonobese, insulin-sensitive PCOS/PMOS populations, and preclinical models to distinguish core pathophysiology from secondary metabolic effects.

#### Chronic inflammation, oxidative stress, and mitochondrial dysfunction

Low-grade systemic inflammation in PCOS/PMOS (174) contributes to neurodegenerative vulnerability (31). As noted above, the level of proinflammatory cytokines, including IL-6 correlate with APCS, a protein biomarker observed in AD in PCOS/PMOS (20), mirroring the bidirectional inflammation–amyloid interactions in AD. In turn, oxidative stress, an early AD event detectable at mild cognitive impairment (37), is driven by iron dyshomeostasis (175), Aβ-mediated generation of hydrogen peroxide and hydroxyl radicals (176), and Aβ-metal coordination (177), impairing essential cellular processes (176) and inducing tau hyperphosphorylation (38).

Mitochondria are both sources and targets of oxidative damage, creating cycles of energy deficit and apoptosis (178). Oxidative stress is elevated in PCOS/PMOS and contributes to its higher incidence of mitochondrial dysfunction (179). However, most evidence for oxidative stress in PCOS/PMOS derives from peripheral biomarkers and metabolically heterogeneous populations. As such, while systemic oxidative stress in PCOS/PMOS may plausibly prime the brain for vulnerability to AD-related cascades, particularly in the presence of obesity or IR, direct evidence linking oxidative stress in normal-weight PCOS/PMOS to progressive neurodegeneration remains limited.

Aβ binds mitochondrial membranes leading to bioenergetic failure and promoting neuronal death (180). A central mechanism involves inhibition of the electron transport chain, particularly complex IV, reducing ATP production and impairing glucose metabolism, and diminished ATP production (181). Impaired mitochondrial biogenesis exacerbates this deficit (182).

In PCOS/PMOS, mitochondrial dysfunction involves altered structure, impaired dynamics, loss of membrane potential, compromised oxidative phosphorylation, and impaired mitophagy (183-185). While shared mitochondrial abnormalities highlight a convergent pathophysiological endpoint characterized by chronic energy deficit and heightened apoptotic vulnerability (148), mitochondrial dysfunction in PCOS/PMOS is strongly influenced by metabolic comorbidities.

#### Autophagy dysregulation

Autophagy plays a stage-dependent role in AD with early compensatory upregulation facilitating clearance of damaged organelles and Aβ (124). Reactive oxygen species can promote autophagic removal of misfolded Aβ, a phenomenon observed in brain regions vulnerable to AD pathology (185). As disease progresses, however, lysosomal dysfunction leads to collapse of autophagic flux, exacerbating proteostasis failure and the accumulation of toxic species (186).

Preclinical evidence indicates IR, a core PCOS/PMOS feature, negatively regulates autophagy, suggesting the chronic metabolic milieu of PCOS/PMOS may establish impaired cellular clearance. This hypothesis is biologically plausible but not empirically established. Current evidence is indirect, and it remains unknown whether autophagy is disrupted in metabolically healthy PCOS/PMOS or contributes to long-term cognitive decline.

#### Dysregulation of tryptophan–kynurenine metabolism

Tryptophan metabolism through the KP pathway represents a biochemical interface between inflammation, metabolism, and neurodegeneration. A shared feature of both AD and PCOS/PMOS is overactivation of this pathway, largely driven by inflammatory signaling (187). This shift alters the balance between neuroprotective metabolites such as kynurenic acid (KYNA) and neurotoxic compounds including quinolinic acid (QA) (148).

In AD, QA localizes to senile plaques (188), its production is promoted by Aβ (189), and it contributes to tau hyperphosphorylation (190, 191). In PCOS/PMOS, elevated circulating KP, KYNA, and QA are reported (192), with associations to androgen levels (193). However, KP overactivation is strongly influenced by obesity, IR, and systemic inflammation (194), all of which are prevalent but not universal in PCOS/PMOS. Therefore, current evidence supports KP dysregulation as a shared metabolic-inflammatory pathway rather than a PCOS/PMOS-specific mechanism. Whether KP alterations persist in normal-weight, metabolically healthy PCOS/PMOS, or directly contribute to central neurotoxicity remains unknown.

### Implications for clinical practice and research

Current evidence does not support routine cognitive screening or dementia risk counselling specifically for PCOS/PMOS patients. Clinical management should prioritize optimization of metabolic health through lifestyle modification and insulin sensitizers, for which established indications exist (195-197); however, whether such strategies confer brain benefits remains unproven (20, 30, 32, 48-50). Intervention data examining cognitive outcomes remain limited and inconsistent and well-designed RCTs with standardized cognitive endpoints, adequate metabolic phenotyping, and longer follow-up are required before recommending PCOS/PMOS-specific treatments for neuroprotection. Until such data are available, neurological benefits of PCOS/PMOS-directed therapies remain speculative.

### Future directions and research gaps

Despite growing knowledge, critical gaps persist. First, it remains unknown whether PCOS/PMOS independently increases AD risk or whether observed associations are mediated through IR, inflammation, and vascular dysfunction. Large-scale, prospective cohort studies spanning the reproductive lifespan are urgently needed. Second, substantial gaps exist in understanding shared molecular mechanisms. Genetic overlap between PCOS/PMOS and AD remains undefined, and the roles of miRNA–mRNA networks (64), epigenetic modifications (101), and metabolic signaling pathways require direct investigation. Finally, whether PCOS/PMOS management can meaningfully alter long-term cognitive trajectories remains unanswered. RCTs with cognitive outcomes as primary endpoints are essential to move beyond associative evidence.

## Conclusions

This review synthesizes evidence linking PCOS/PMOS to cognitive alterations and AD-relevant biological pathways, primarily through IR, inflammation, and hormonal dysregulation. Neuroimaging and biomarker studies demonstrate brain-related changes in PCOS/PMOS, yet evidence for increased dementia risk remains inconsistent and cognitive benefits of PCOS/PMOS-specific interventions are lacking. Current evidence indicates that any association between PCOS/PMOS and AD is likely mediated by metabolic comorbidities rather than reflecting an independent PCOS/PMOS effect. Clinical management should prioritize metabolic health, while well-designed prospective, phenotype-stratified mechanistic studies and RCTs with cognitive endpoints are needed to clarify long-term neurological outcomes.

## Data Availability

The data generated by this review will be shared upon reasonable request.
